# Supplementary material for: Dietary Habits and Gut Microbiota in Healthy Adults: Focusing on the Right Diet. A Systematic Review
Source: Int J Mol Sci. 2021 Jun 23;22(13):6728. doi: 10.3390/ijms22136728 (PMC8269086; doi:10.3390/ijms22136728)
Supplement: Supplementary file 1 [file ijms-22-06728-s001.zip › Supplementary table S1.pdf]

Supplementary Table S1 List of excluded full text studies.

Author, year	Title	Reason for exclusion
Chambers, 2019 [1]	Dietary supplementation with inulin-propionate ester or inulin improves insulin sensitivity in adults with overweight and obesity with distinct effects on the gut microbiota, plasma metabolome and systemic inflammatory responses: a randomised crossover trial	Not dietary regimen specified but inulin derivates. Moreover, the population study is not heatlhy adults
Cox , 2020 [2]	Effects of Low FODMAP Diet on Symptoms, Fecal Microbiome, and Markers of Inflammation in Patients With Quiescent Inflammatory Bowel Disease in a Randomized Trial	Low-FODMAP diet not clearly identified and also population study of patients affected by IBD
Depommier, 2019 [3]	Supplementation with *Akkermansia muciniphila* in overweight and obese human volunteers: a proof-of-concept exploratory study	Single supplementation not identified as specific diet
Frost, 2019 [4]	A structured weight loss program increases gut microbiota phylogenetic diversity and reduces levels of *Collinsella* in obese type 2 diabetics: A pilot study	Population study of patients affected by diabetes
Gutierrez-Repiso, 2019 [5]	Effect of Synbiotic Supplementation in a Very-Low-Calorie Ketogenic Diet on Weight Loss Achievement and Gut Microbiota: A Randomized Controlled Pilot Study	Very-low calorie ketogenic diet difficult to identify and to reproduce
Watson, 2017 [6]	A randomised trial of the effect of omega-3 polyunsaturated fatty acid supplements on the human intestinal microbiota.	Omega-3 supplements not recognised as specific diet regimen
Ross, 2013 [7]	A Whole-Grain–Rich Diet Reduces Urinary Excretion of Markers of Protein Catabolism and Gut Microbiota Metabolism in Healthy Men after One Week	Difficult to assess whole-grain-rich diet and short period of exposition
Vuholm, 2017 [8]	Whole-Grain Rye and Wheat Affect Some Markers of Gut Health without Altering the Fecal Microbiota in Healthy Overweight Adults: A 6-Week Randomized Trial	Difficult dietary regimen and obese adults
Bonder, 2016 [9]	The influence of a short-term gluten-free diet on the human gut microbiome	Not a diet eligible for healthy adults and short time of exposition
De Palma, 2009 [10]	Effects of a gluten-free diet on gut microbiota and immune function in healthy adult human subjects	Only ten participants for one month of exposition to a diet not clearly eligible for healthy adults. Not completely within the time period considered.

References

1. Chambers, E.S.; Byrne, C.S.; Morrison, D.J.; Murphy, K.G.; Preston, T.; Tedford, C.; Garcia-Perez, I.; Fountana, S.; Serrano-Contreras, J.I.; Holmes, E.; Reynolds, C.J.; Roberts, J.F.; Boyton, R.J; Altmann, D.M.; McDonald, J.A.K; Marchesi, J.R.; Akbar, A.N.; Riddell, N.E.; Wallis, G.A; Frost, G.S. Dietary supplementation with inulin-propionate ester or inulin improves insulin sensitivity in adults with overweight and obesity with distinct effects on the gut microbiota, plasma metabolome and systemic inflammatory responses: a randomised cross-over trial. *Gut* **2019,** 68(8):1430-1438. doi: 10.1136/gutjnl-2019-318424.
2. Cox, S.R.; Lindsay, J.O.; Fromentin, S.; Stagg, A.J.; McCarthy, N.E.; Galleron, N.; Ibraim, S.B.; Roume, H.; Levenez, F.; Pons, N.; Maziers, N.; Lomer, M.C.; Ehrlich, S.D.; Irving, P.M.; Whelan K. Effects of Low FODMAP Diet on Symptoms, Fecal Microbiome, and Markers of Inflammation in Patients With Quiescent Inflammatory Bowel Disease in a Randomized Trial. *Gastroenterology* **2020,** 158(1):176-188.e7. doi: 10.1053/j.gastro.2019.09.024.

3. Depommier, C.; Everard, A.; Druart, C.; Plovier, H.; Van Hul, M.; Vieira-Silva, S.; Falony, G.; Raes, J.; Maiter, D.; Delzenne, N.M.; de Barsy, M.; Loumaye, A.; Hermans, M.P.; Thissen, J.P.; de Vos, W.M.; Cani, P.D. Supplementation with Akkermansia muciniphila in overweight and obese human volunteers: a proof-of-concept exploratory study. *Nat Med* **2019,** 25(7):1096-1103. doi: 10.1038/s41591-019-0495-2.
4. Frost, F.; Storck, L.J.; Kacprowski, T.; Gärtner, S.; Rühlemann, M.; Bang, C.; Franke, A.; Völker, U.; Aghdassi, A.A.; Steveling, A.; Mayerle, J.; Weiss, F.U.; Homuth, G.; Lerch, M.M. A structured weight loss program increases gut microbiota phylogenetic diversity and reduces levels of Collinsella in obese type 2 diabetics: A pilot study. *PLoS One* **2019,** 14(7):e0219489. doi: 10.1371/journal.pone.0219489.
5. Gutiérrez-Repiso, C.; Hernández-García, C.; García-Almeida, J.M.; Bellido, D.; Martín-Núñez, G.M.; Sánchez-Alcoholado, L.; Alcaide-Torres, J.; Sajoux, I.; Tinahones, F.J.; Moreno-Indias, I. Effect of Synbiotic Supplementation in a Very-Low-Calorie Ketogenic Diet on Weight Loss Achievement and Gut Microbiota: A Randomized Controlled Pilot Study. *Mol Nutr Food Res* **2019,** 63(19):e1900167. doi: 10.1002/mnfr.201900167.
6. Watson, H.; Mitra, S.; Croden, F.C.; Taylor, M.; Wood, H.M.; Perry, S.L.; Spencer, J.A.; Quirke, P.; Toogood, G.J.; Lawton, C.L.; Dye, L.; Loadman, P.M.; Hull, M.A. A randomised trial of the effect of omega-3 polyunsaturated fatty acid supplements on the human intestinal microbiota. *Gut* **2018,** 67(11):1974-1983. doi: 10.1136/gutjnl-2017-314968.
7. Ross, A.B.; Pere-Trépat, E.; Montoliu, I.; Martin, F.P.; Collino, S.; Moco, S.; Godin, J.P.; Cléroux, M.; Guy, P.A., Breton, I.; Bibiloni, R.; Thorimbert, A.; Tavazzi, I.; Tornier, L.; Bebuis, A.; Bruce, S.J.; Beaumont, M.; Fay, L.B.; Kochhar, S. A whole-grain-rich diet reduces urinary excretion of markers of protein catabolism and gut microbiota metabolism in healthy men after one week. *J Nutr* **2013,** 143(6):766-73. doi: 10.3945/jn.112.172197.
8. Vuholm, S.; Nielsen, D.S.; Iversen, K.N.; Suhr, J.; Westermann, P.; Krych, L.; Andersen, J.R.; Kristensen, M. Whole-Grain Rye and Wheat Affect Some Markers of Gut Health without Altering the Fecal Microbiota in Healthy Overweight Adults: A 6-Week Randomized Trial. *J Nutr* **2017,** 147(11):2067-2075. doi: 10.3945/jn.117.250647.
9. Bonder, M.J.; Tigchelaar, E.F.; Cai, X.; Trynka, G.; Cenit, M.C.; Hrdlickova, B.; Zhong, H.; Vatanen, T.; Gevers, D.; Wijmenga, C.; Wang, Y.; Zhernakova, A. The influence of a short-term gluten-free diet on the human gut microbiome. *Genome Med* **2016**, 8(1):45. doi: 10.1186/s13073-016-0295-y.
10. De Palma, G.; Nadal, I.; Collado, M.C.; Sanz Y. Effects of a gluten-free diet on gut microbiota and immune function in healthy adult human subjects. *Br J Nutr* **2009,** 102(8):1154-60. doi: 10.1017/S0007114509371767.
