# Supplementary material for: Dietary Habits and Gut Microbiota in Healthy Adults: Focusing on the Right Diet. A Systematic Review
Source: Int J Mol Sci. 2021 Jun 23;22(13):6728. doi: 10.3390/ijms22136728 (PMC8269086; doi:10.3390/ijms22136728)
Supplement: Supplementary file 1 [file ijms-22-06728-s001.zip › Supplementary table S2.pdf]

Supplementary Table S2 Cochrane Collaboration tool for assessing risk of bias in randomized controlled trials included in the study.

Study	Random sequence generation (*selection bias*)	Allocation concealment (*selection bias*)	Blinding of participants and personnel (*performance bias*)	Blinding of outcome assessment (*detection bias*) (patient-reported outcomes)	Incomplete outcome data addressed (*attrition bias*) (short-term outcomes 2-6 wks)	Incomplete outcome (*attrition bias*) (Longer-term outcomes > 6 wks)	Selective reporting (*reporting bias*)	Other potential threats to validity
Pagliai G, 2019 [29,30]	High	Low	Low	Low	Not applicable	Low	Low	Low
Wan Y, 2019 [33,34]	Low	Low	Low	Low	Not applicable	Low	Low	Low
Ghosh TS, 2020 [35,36]	Low	Low	Low	Low	Not applicable	Low	Low	Low
