# Supplementary material for: Dietary Habits and Gut Microbiota in Healthy Adults: Focusing on the Right Diet. A Systematic Review
Source: Int J Mol Sci. 2021 Jun 23;22(13):6728. doi: 10.3390/ijms22136728 (PMC8269086; doi:10.3390/ijms22136728)
Supplement: Supplementary file 1 [file ijms-22-06728-s001.zip › Supplementary table S3 .pdf]

Supplementary Table S3. Newcastle–Ottawa scale adapted for cross-sectional studies.

	Selection				Comparability	Outcome		Total
Study	Representa-tiveness of sample	Sample size	Non-respondents	Ascertainment of the exposure (risk factor)	Comparability of subjects in different outcome group based on the study design or analysis	Assessment of outcome	Statistical test	
Ferrocino, I [19]	●	●	-	●	-	●●	●	6/10
De Filippis F, 2016 [20]	●	●	-	●	-	●●	●	6/10
Wu GD, 2016 [21]	-	-	-	●●	-	●●	●	5/10
Gùtierrez-Diàz I, 2016 [22]	-	-	-	●●	-	●●	●	5/10
Gùtierrez-Diàz I, 2017 [23]	●	●	-	●●	-	●●	●	7/10
Federici E, 2017 [24]	-	-	-	●	-	●●	●	4/10
Franco-de-Moraes AC, 2017 [25]	●	●	-	●	-	●●	●	6/10
Mitsou E.K, 2017 [26]	●	●	-	●●	-	●●	●	7/10
Losasso C, 2018 [27]	●	●	-	●●	-	●●	●	7/10
Trefflich I, 2019 [18]	●	●	-	●	-	●●	●	6/10
Wang F, 2019 [31]	-	-	-	●●	-	●●	●	5/10
Stefani, S 2018 [28]	-	●	-	●	-	●●	●	4/10
Luisi MLE, 2019 [32]	-	-	-	●	-	●●	●	4/10
